# 

**Published:** 1892-01-09

**Authors:** 


					MNBORNPS
for INFANTS ; !
ISINGLASS
and INVALIDS.
No. 2W. Vol. XI.] Saturday,
January 9th, 1892.
[One Penny.

				

